# *QuickStats:* Percentage* of Children and Adolescents Aged 4–17 Years with Serious Emotional or Behavioral Difficulties,^†^ by Sex and Urbanization Level^§^ — National Health Interview Survey, 2016–2018^¶^

**DOI:** 10.15585/mmwr.mm6910a6

**Published:** 2020-03-13

**Figure:**
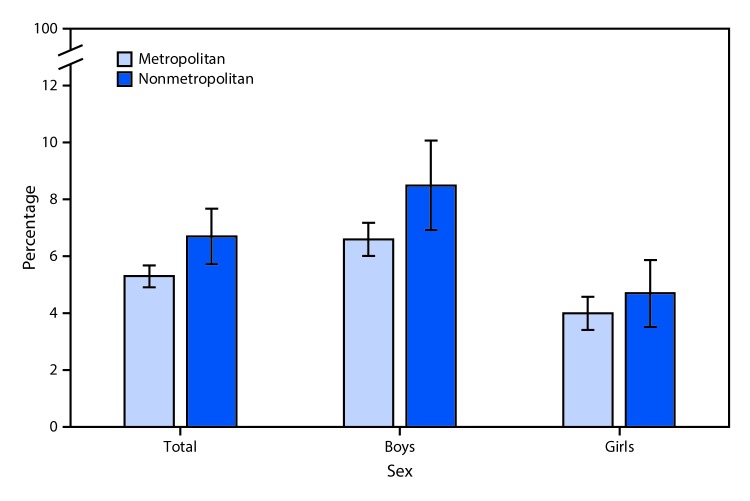
During 2016–2018, the percentage of children and adolescents aged 4–17 years with serious emotional or behavioral difficulties was higher among those living in nonmetropolitan areas (6.7%) than among those living in metropolitan areas (5.3%). Among boys, those living in nonmetropolitan areas (8.5%) were more likely to have serious emotional or behavioral difficulties than those living in metropolitan areas (6.6%), but the difference among girls was smaller and not significant. Among children and adolescents living in either metropolitan or nonmetropolitan areas, boys were more likely than girls to have serious emotional or behavioral difficulties.

**Source:** National Health Interview Survey, 2016–2018. https://www.cdc.gov/nchs/nhis/index.htm.

